# Effect of Pb_3_O_4_ nanocomposite on the structural, optical, and radiation shielding properties of PVA Films

**DOI:** 10.1038/s41598-025-22740-6

**Published:** 2025-10-31

**Authors:** Yasmin Hamed, Khaled Salahel Din, S. Harb, Sahar Elnobi

**Affiliations:** Physics Department, Faculty of Science, South Valley University, Qena, 83523 Egypt

**Keywords:** PVA, Pb_3_O_4_ nanocomposite, Optical properties, Gamma ray shielding, Attenuation coefficient, Materials science, Nanoscience and technology, Optics and photonics, Physics

## Abstract

This study investigated the effect of lead tetroxide nanoparticles (Pb_3_O_4_ NPs) on the properties of polyvinyl alcohol (PVA). PVA films were prepared by a conventional casting method, with varying concentrations of Pb_3_O_4_ NPs (0, 2, 4, 6, 7, and 9 wt %). A significant increase in the crystallinity of the PVA film with increasing Pb_3_O_4_ NPs concentrations and the uniform distribution of Pb_3_O_4_ NPs on the surface of the films were demonstrated using X-ray diffraction (XRD) analysis and scanning electron microscopy (SEM), respectively. Optical characterizations revealed increased light absorption and decreased light transmittance with increasing Pb_3_O_4_ concentration. Among the significant optical results, the band gap energy of the samples decreased from 4.95 to 3.77 eV for pure PVA and PVA/9% Pb_3_O_4_. The radiation blocking capabilities of the PVA films were investigated experimentally using a NaI (Tl) detector and theoretically using Phy-X/PSD software in the gamma-ray energy range (0.511–1.332) MeV. The experimental and theoretical results were consistent, highlighting that higher concentrations of Pb_3_O_4_ improve the effectiveness of PVA films in reducing gamma ray penetration. Thus, the control of the filler concentrations to enhance the shielding properties will open up a new strategy to synthesize novel materials for radiation applications.

**Supplementary Information:**

The online version contains supplementary material available at 10.1038/s41598-025-22740-6.

## Introduction

Polymers are large molecules composed of small structural units called monomers that repeat in the polymer chain. Polymers are used in many industries because they are inexpensive, lightweight, flexible, and easy to process^1–3^. PVA, a water-soluble polymer with superior mechanical qualities, high dielectric strength, great chemical stability, and non-toxicity, is the most often used synthetic polymer. PVA can also be easily chemically modified to suit a variety of functional applications^3–5^. All of this makes PVA a very suitable material for use in radiation shielding compounds when blended with high-density fillers^6,7^.

Radiation is defined as energy emitted from the atoms of unstable elements, and it has the potential to pass through a variety of materials. Based on its capacity to ionize materials, radiation can be divided into two primary groups: (i) ionizing and (ii) nonionizing. The energy of nonionizing radiation is insufficient to expel electrons from the atom and create ions, such as visible light, microwaves, radio waves, infrared, and sunlight^8,9^. Ionizing radiation is electromagnetic radiation with a higher energy than nonionizing radiation, which creates positively charged ionized atoms and negatively charged free electrons. It can also eject electrons from atoms, and it consists of charged particles or electromagnetic radiation such as alpha particles, beta particles, gamma radiation, X-rays, and neutrons^9^. Among these, gamma rays are the most dangerous due to their high penetrating power and their ability to cause severe biological damage, including DNA damage and cancer^10,11^. Excessive exposure to ionizing radiation can pose serious health risks^12^.Therefore, there is a need to identify effective radiation shielding materials that can attenuate radiation intensity to safe levels. However, gamma rays must be shielded with great intensity, especially in medical, industrial, and nuclear research settings^13,14^. Radiation shielding methods are based on three main principles: (i) Minimizing exposure time, (ii) Increasing distance from the radiation source, and (iii) Using a shielding material that can absorb or attenuate radiation^15,16^. The choice of material for shielding against X-rays and gamma rays depends primarily on its density and atomic number—the higher the atomic number and density, the greater the attenuation^17–19^.

Lead and concrete have traditionally been used due to their weight and ability to trap radiation via the photoelectric effect and Compton scattering^20,21^. However, their major drawbacks include toxicity, heavy weight, cost, and fragility, which limit their everyday application^22^. Due to these drawbacks, there has recently been growing interest in the use of polymer-based composites as radiation shields. Polymers are light, flexible, and easy to process, but they lack the density required to absorb gamma rays and are therefore insufficient to attenuate gamma rays alone. To counter this, polymers are typically loaded with high-density metal oxides^23^. For example, if PVA is doped with Pb_3_O_4_ (our present study), a high-density material, the resulting composite can provide an effective gamma ray shield without compromising its low weight and flexibility^24^. Several previous studies have evaluated the potential uses of polymeric composites for radiation shielding. Almurayshid et al.^25^, they demonstrated that HDPE composites blended with tungsten and molybdenum particles showed that 15% W increased the linear attenuation coefficient (LAC) to 0.134 cm^-1^ at 0.662 MeV, but the higher filler loading ratios came at the expense of mechanical flexibility. Ahmed M. Al-Khatib et al.^26^, in their study of polypropylene (PP) nanocomposites reinforced with nano-PbO, they found that 50% nano-PbO had a higher LAC of 5.8793 cm^-1^ at 0.060 MeV than micro-PbO due to improved particle dispersion despite increased stiffness. In addition, Naima and Kazem investigated polypropylene doped with cadmium oxide (CdO) and iron oxide (Fe₂O₃) and concluded that these impurities increase protection against gamma rays and neutrons^27^.

Furthermore, one of the most significant problems associated with the use of Pb_3_O_4_ as a radiation shield is its high toxicity, which poses serious environmental and health risks when exposed via inhalation or skin contact. To avoid this (present study), Pb_3_O_4_ NPs were encapsulated within PVA matrix using traditional casting method to create flexible and well-organized films, rather than being directly manipulated. The impact of Pb_3_O_4_ NPs concentration on structural, optical properties, and gamma radiation shielding of the PVA polymer matrix were explored. These films will be a promising material for flexible and wearable Shielding, nuclear industry, and medical radiation application.

## Materials and methods

### Sample preparation

**Scheme 1:**
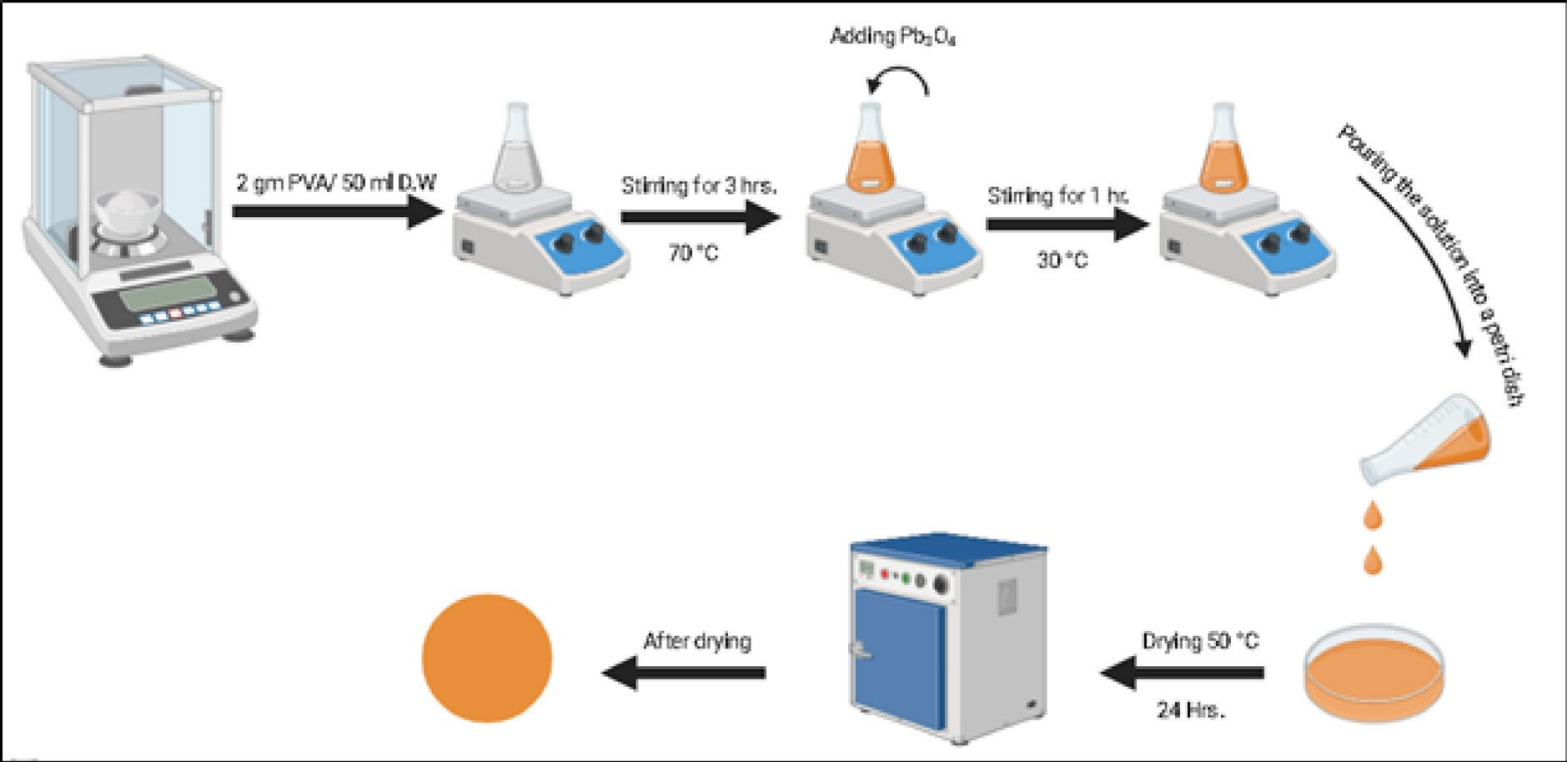
Preparation of the PVA/Pb₃O₄ films by traditional casting method.

PVA and Pb₃O₄ were procured from LOBA Chemie. Prepared polymer samples consisting of PVA with molecular weights of 125000 g/mol as a basic component of the polymer matrix. Pb₃O₄ powder purity was 85% with average crystallite size of 47.05 nm as determined by Scherrer equation analysis has been used as filler. The materials were provided by Alpha Chemieka, India. Solution casting was applied for fabricating PVA/ Pb₃O₄ nanocomposites and pure PVA films. In the case of composites, 2 grams of PVA were dissolved in 50 ml of distilled water at 70°C under continuous stirring for 3 hours to get a homogeneous solution. Different weights of 0.04, 0.08, 0.12, 0.16, and 0.2 grams of Pb₃O₄ were then added to the respective PVA solutions. The mixtures were stirred vigorously for 1 hour for uniform dispersion of Pb₃O₄ in the polymer matrix. The solutions were then cast on clean Petri dishes and allowed to dry slowly at 50°C for 24 hours to form films as shown in Scheme 1. Thickness measurements were performed using Image J software for SEM cross-sectional image to obtain five values from spatially distributed locations on each film. The reported value of 250 ± 3 μm represents the mean values with standard deviation, confirming minimal spatial variation and providing a statistically reliable baseline for optical and radiation protection properties.

Based on the weights incorporated, the weight concentrations of Pb₃O₄ within the composites were 0, 2, 4, 6, 7, and 9% by weight, labeled from S1 to S6, respectively, as described in Schematic S1. The following equation was used to determine the weight percentage of Pb_3_O_4_ in the polymer matrix^28^:

1 $${{\varvec{W}}}_{{{\varvec{P}}{\varvec{b}}}_{3}{{\varvec{O}}}_{4}}\left(\boldsymbol{\%}\right)=\frac{{{\varvec{W}}}_{{{\varvec{P}}{\varvec{b}}}_{3}{{\varvec{O}}}_{4}}}{{{\varvec{W}}}_{{\varvec{P}}{\varvec{V}}{\varvec{A}}}+{{\varvec{W}}}_{{{\varvec{P}}{\varvec{b}}}_{3}{{\varvec{O}}}_{4}}}\boldsymbol{*}100$$

where, ${{\varvec{W}}}_{{{\varvec{P}}{\varvec{b}}}_{3}{{\varvec{O}}}_{4}}$ is the weight of Pb_3_O_4_ in grams and ${{\varvec{W}}}_{{\varvec{P}}{\varvec{V}}{\varvec{A}}}$ is the weight of PVA in grams.

### Characterizations of PVA/Pb₃O₄ films

The crystal structures of all materials were examined by X-ray diffraction (XRD) with a Bruker D8 Advance apparatus, employing monochromatic Cu-Kα radiation (λ = 1.54056 Å) at 40 kV and 60 mA, within a 2θ range of 10–70 degrees. Fourier-transform infrared spectroscopy (FT-IR, Jasco Model 6100, resolution 1 cm⁻^1^, 400–3600 cm⁻^1^ range) was utilized to examine the chemical structure of PVA/Pb₃O₄ films. Scanning electron microscopy (SEM, Quanta FEG250) coupled with energy-dispersive X-ray spectroscopy (EDS) for morphological investigation and elemental quantification, respectively; and UV–Vis-NIR spectroscopy (JASCO 670, 200–2000 nm range) for optical property analysis.

The bandgap energy of a material, defined as the energy required for electrons to move from the valence band to the conduction band, were calculated using the Tauc formula, which is a formula that connects the material’s energy band and absorption coefficient (α)^29^:

2 $${(\alpha h\nu )}^{n}=A\left(h\nu -Eg \right)$$

where, $h\nu$ is photon energy, A is a constant ,and $n$ is a constant that depends on the nature of the electronic transitions from the valence band to the conduction band, where $n=\frac{1}{2 }or \frac{3}{2}$ for direct transitions, while $n=2 or 3$ for indirect transitions whether they are allowed or forbidden, respectively^7,29,30^.

### Shielding properties

**Scheme 2:**
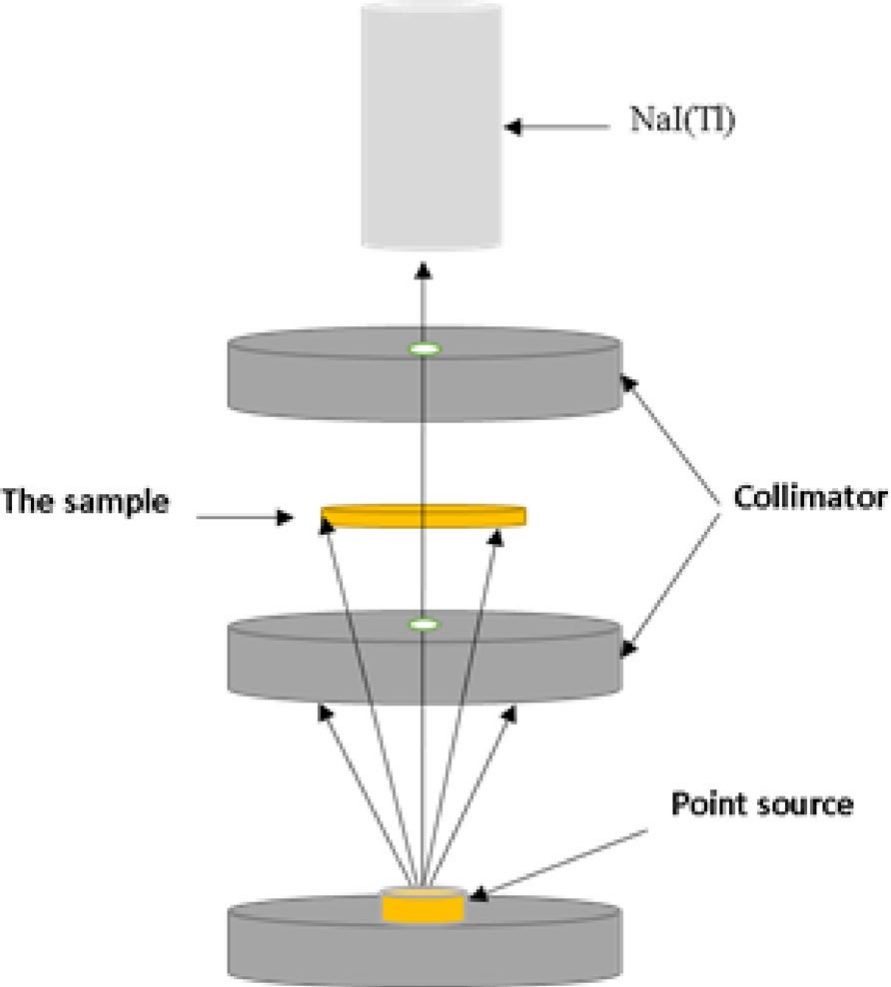
The geometric arrangement of the system.

A calibrated (3 × 3 inch) NaI (Tl) detector connected to a multichannel analyzer was utilized to assess the shielding properties of the prepared PVA and PVA/Pb₃O₄ films. The samples were exposed to gamma ray photons emitted from radioactive point sources (Na-22, Cs-137, and Co-60) in the energy range of 0.511 to 1.332 MeV, as illustrated in Scheme 2. Using Genie 2000 software, the spectrum of gamma photons transmitted through the samples and captured by the detector was recorded after a sufficient time that minimizes the statistical error. For each gamma photons energy and PVA and PVA/Pb₃O₄ films, the net count under peaks of the spectrum was used to calculate the shielding parameters as describe below:

#### Linear attenuation coefficient (LAC)

The LAC defines as a measure of the ability of a material to attenuate the intensity of radiation passing through a given thickness of material. It is expressed in units of cm⁻^1^ and depends on the energy of the incident radiation, the density (ρ), and the atomic number (Z) of the material. The LAC can have calculated from the Beer-Lambert Eq. ^31^:

3 $$I={I}_{o}{e}^{-LAC X}$$

where $, {I}_{o}$ denotes the intensity of incident gamma ray without any absorbing medium, $I$ represents the transmitted intensity after passing through absorber thickness (X), and LAC is the linear attenuation coefficient^27,32^.

#### Mass attenuation coefficient (MAC)

The MAC in cm^2^/g expresses the ability of a material to attenuate radiation per unit mass, and it is calculated from the linear attenuation coefficient divided by the density of the material (ρ) g/cm^3^^33,34^ The mass attenuation coefficient can be calculated from the equation

4 $$MAC=\frac{{\varvec{L}}{\varvec{A}}{\varvec{C}}}{{\varvec{\rho}}} {cm}^{2}/g$$

The error in the MAC measurements was calculated from the following Eq. ^35^:

5 $$\Delta MAC=\frac{1}{\rho x}\{{\left(\frac{\Delta Io}{Io}\right)}^{2}+{\left(\frac{\Delta I}{I}\right)}^{2}+{\left[\text{ln}\left(\frac{Io}{I}\right)\right]}^{2}{\left[{\left(\frac{\Delta \rho }{\rho }\right)}^{2}+{\left(\frac{\Delta x}{x}\right)}^{2}\right]\}}^\frac{1}{2}$$

#### Half value layer (HVL)

The HVL represents the material thickness needed to attenuate radiation intensity to 50% of its original value and is calculated using the Eq. ^10,25,36^:

6 $$\text{HVL }= \frac{\text{ln}2}{\text{LAC}}$$

#### Mean free path (MFP):

The average distance between two consecutive gamma photon interactions with the absorbing material is known as the MFP , and it may be computed using the formula^35^:

7 $$\text{MFP}=\frac{1}{\text{LAC}}$$

To check the validity of the experimental results, the linear and/or mass attenuation coefficients (MACs) were calculated theoretically by the Phy-X/PSD software^37,38^. The chemical compositions of each PVA sample were entered into the software to get the theoretical data.

The deviations (%) between experimental results and Phy-X values were measured using the next formula^39^:

8 $${\text{Dev }}\left( \% \right) = \left| {\frac{{{\text{MAC}}\left( {{\text{Phy}} - X} \right) - {\text{MAC}}\left( {{\text{Exp}}} \right)}}{{{\text{MAC}}\left( {{\text{Phy}} - X} \right)}}*100} \right|$$

## Results and discussions

### Structural properties of PVA/Pb_3_O_4_ nanocomposite films

#### X-ray diffraction (XRD) analysis

**Fig. 1:**
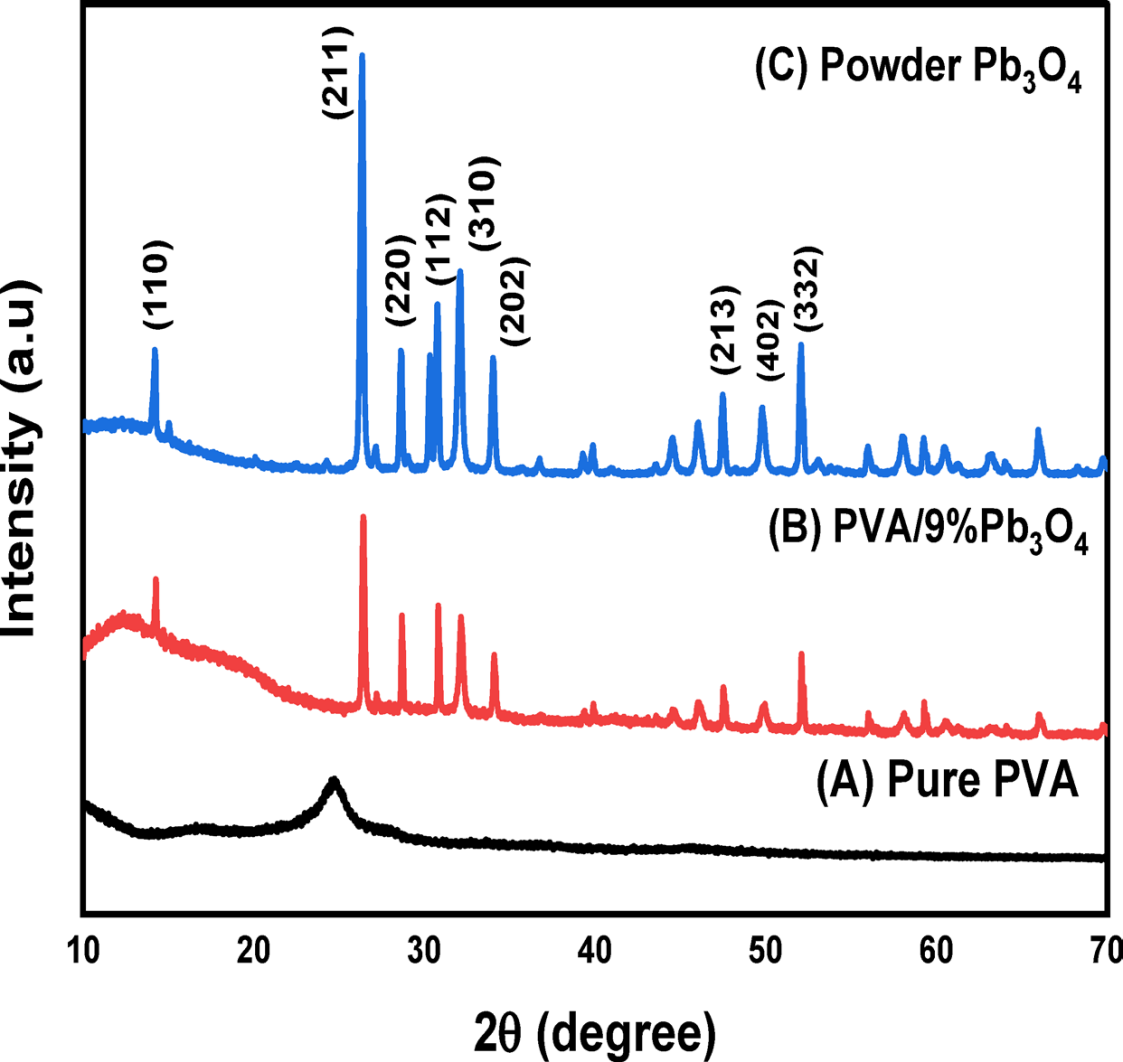
XRD patterns of pure PVA, PVA/ Pb₃O₄film and Pb₃O₄ powder.

XRD analysis (Fig. 1) reveals distinct structural characteristics: Pure PVA (a) exhibits a broad peak at 2θ ≈ 19.7°, indicative of its semi-crystalline/amorphous nature arising from disordered polymer chains and intermolecular interactions^40–42^. The PVA/9% Pb₃O₄ composite film (b) shows sharp diffraction peaks corresponding to crystalline Pb₃O₄ (e.g., 25.5° (110), 32.5° (211), 36.5° (220), 41.5° (112), 42.5° (310), 46.5° (202)), confirming the preservation of Pb₃O₄ crystallinity within the amorphous PVA matrix. The absence of PVA’s characteristic peak and reduced intensity of Pb₃O₄ peaks relative to pure powder (c) suggests (i) Enhanced overall crystallinity due to Pb₃O₄ incorporation ^28^, (ii) Effective dispersion of Pb₃O₄ crystallites, (iii) Potential crystallite size reduction, and (iv) Polymer-particle interfacial interactions. The well-defined Bragg reflections in pure Pb₃O₄ powder (c) demonstrate long-range atomic periodicity characteristic of crystalline solids. Scherrer analysis^44,45^ of the dominant peak at 2θ ≈ 26.42° yields a crystallite size of 47.05 nm, confirming nanoscale Pb₃O₄ crystallites with uniform PVA-matrix distribution consistent with microcrystalline composite formation^44,46^.

#### Fourier transform infrared (FT-IR)

**Fig. 2:**
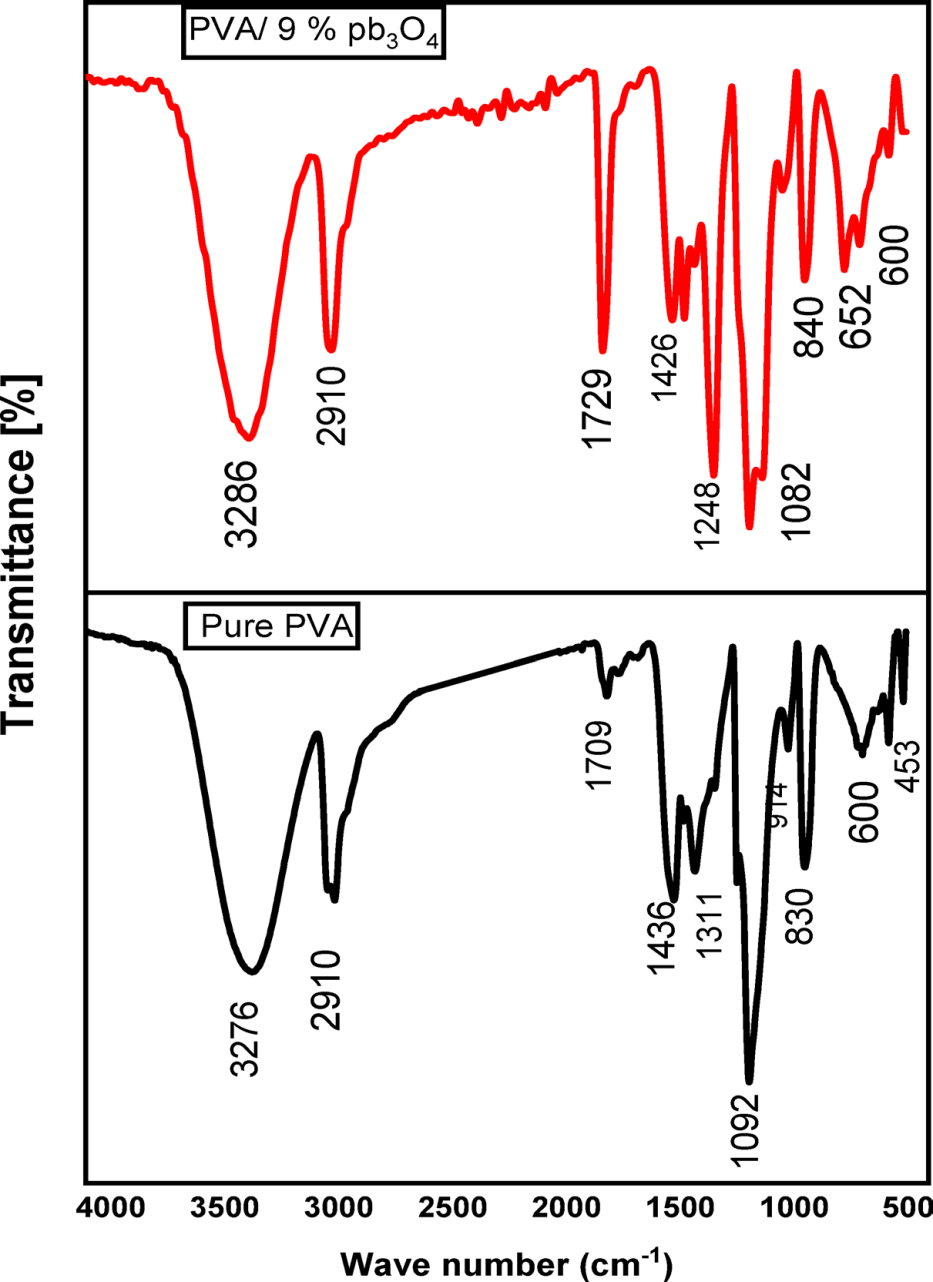
FTIR spectra for pure PVA and PVA/9% Pb₃O₄.

Figure 2 presents the FTIR spectra of as-synthesized pure PVA and PVA/9% Pb₃O₄ composite. The spectrum of pure PVA exhibits characteristic absorption bands: a broad O–H stretching vibration at 3276 cm^-1^, asymmetric CH_2_ stretching at 2910 cm^-1^, a feature at 1709 cm^-1^ indicative of water adsorption, CH_2_ bending at 1436 cm^-1^, O–H bending coupled to CH vibrations at 1311 cm^-1^, a prominent C-O stretching/O–H bending peak at 1092 cm^-1^ (characteristic of amorphous PVA), CH_2_ vibration at 914 cm⁻^1^, and C–C stretching/intrachain vibrations at 830 cm^-1^^47^.Incorporation of Pb₃O₄ induces significant spectral modifications indicative of biphasic interactions. While core PVA peaks persist (e.g., O–H stretch at 3286 cm^-1^, CH_2_ stretch at 2910 cm^-1^, a band at 1729 cm^-1^ potentially from residual C=O/water, CH_2_ bend at 1426 cm⁻^1^), notable shifts and intensity changes occur, including a bathochromic shift of the O- stretch from 3276 to 3286 cm^-1^. The, new bands emerge at lower wavenumbers (840, 652 and 600 cm^-1^), attributable to Pb–O vibrational modes^28,49^. These alterations in wavenumber, intensity, and the appearance of filler-specific peaks provide clear evidence of strong interfacial interactions, likely hydrogen bonding or coordination, between the PVA matrix and Pb₃O₄ particles, confirming successful integration of the filler^50,51^.

#### Scanning electron microscope (SEM) and energy-dispersive X-ray (EDX)

**Fig. 3:**
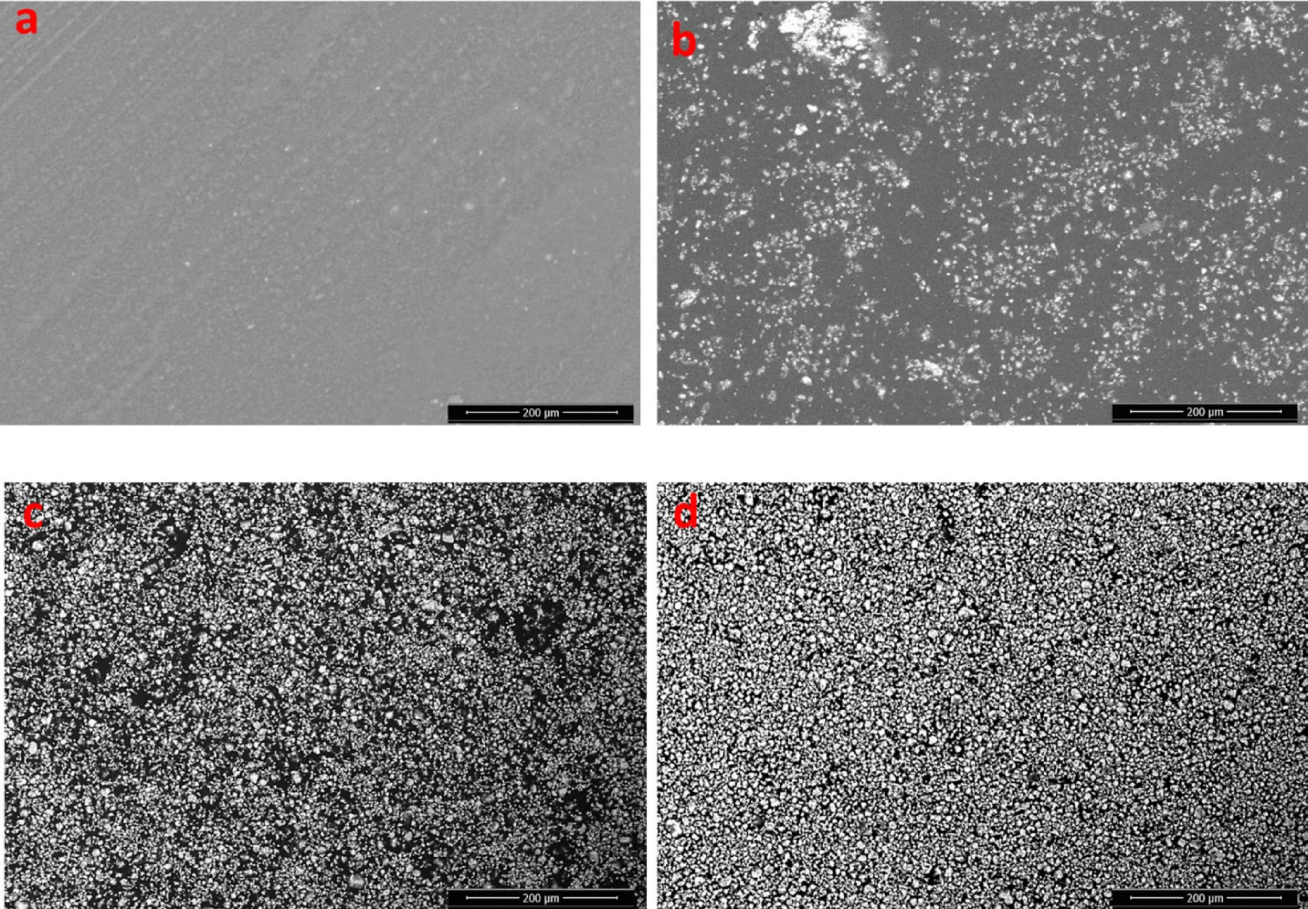
SEM images (**a**) pure PVA, (**b**) PVA/2%Pb₃O₄, (**c**) PVA/6%Pb₃O₄, and PVA/9%Pb₃O₄.

SEM is utilized to examine the surface morphology and microstructural development of pure polyvinyl alcohol (PVA) and PVA/Pb_3_O_4_ composite films. Figure 3a reveals that the pure PVA film possesses a characteristically smooth, homogeneous, and featureless surface, consistent with its amorphous nature and the absence of phase separation or foreign inclusions. The incorporation of Pb₃O₄ particles induces progressive and significant alterations in the surface topography. At a low concentration (2 wt. %, Fig. 3b), the Pb_3_O_4_ particles are present as isolated granular aggregates, uniformly dispersed across the polymer surface. The persistent visibility of the underlying smooth PVA matrix indicates a filler loading below the percolation threshold and suggests good filler-matrix compatibility, which inhibits extensive agglomeration. With increased loading (6 wt %, Fig. 3c), the composite exhibits the onset of particle interconnection, forming an open yet more continuous network. While the distribution remains uniform, the increasing surface coverage by the filler obscures the polymer matrix. At the highest concentration (9 wt %, Fig. 3d), the surface undergoes a complete morphological transformation, dominated by a dense, uniform, and highly interconnected network of Pb_3_O_4_ granules. This percolative structure establishes a distinct topography with clear phase boundaries, which is highly advantageous for enhancing photon interaction mechanisms, such as photoelectric absorption.

EDX analysis corroborates the SEM observations and quantitatively tracks the incorporation of lead. The spectrum of pure PVA (Fig. S1a) displays only carbon (C) and oxygen (O) signals, aligning with its chemical structure (–C_2_H_4_O–). The spectrum for the 2 wt% composite (Fig. S1b) shows the emergence of distinct lead (Pb) peaks, confirming the successful incorporation of the filler, albeit at a low intensity corresponding to the low concentration. The intensity of the Pb signal increases markedly in the 6 wt% sample (Fig. S1c), reflecting the higher filler content. Finally, the spectrum for the 9 wt% composite (Fig. S1d) is dominated by strong Pb and O signals from Pb_3_O_4_, superimposed on the diminished carbon signal from the PVA matrix, visually confirming the surface coverage observed in SEM. These results demonstrate that the PVA matrix provides effective encapsulation for the Pb_3_O_4_ particles. These findings are consistent with contemporary research on using biocompatible polymers to encapsulate toxic functional materials to enhance biosafety in medical and radiological applications^13^. Thus, the developed PVA/Pb_3_O_4_ composite films represent a promising, safe, and effective material for gamma radiation shielding, as evidenced by their optimal morphological homogeneity, complete surface coverage, and verified elemental composition.

### Optical properties of PVA/ Pb_3_O_4_ films

**Fig. 4:**
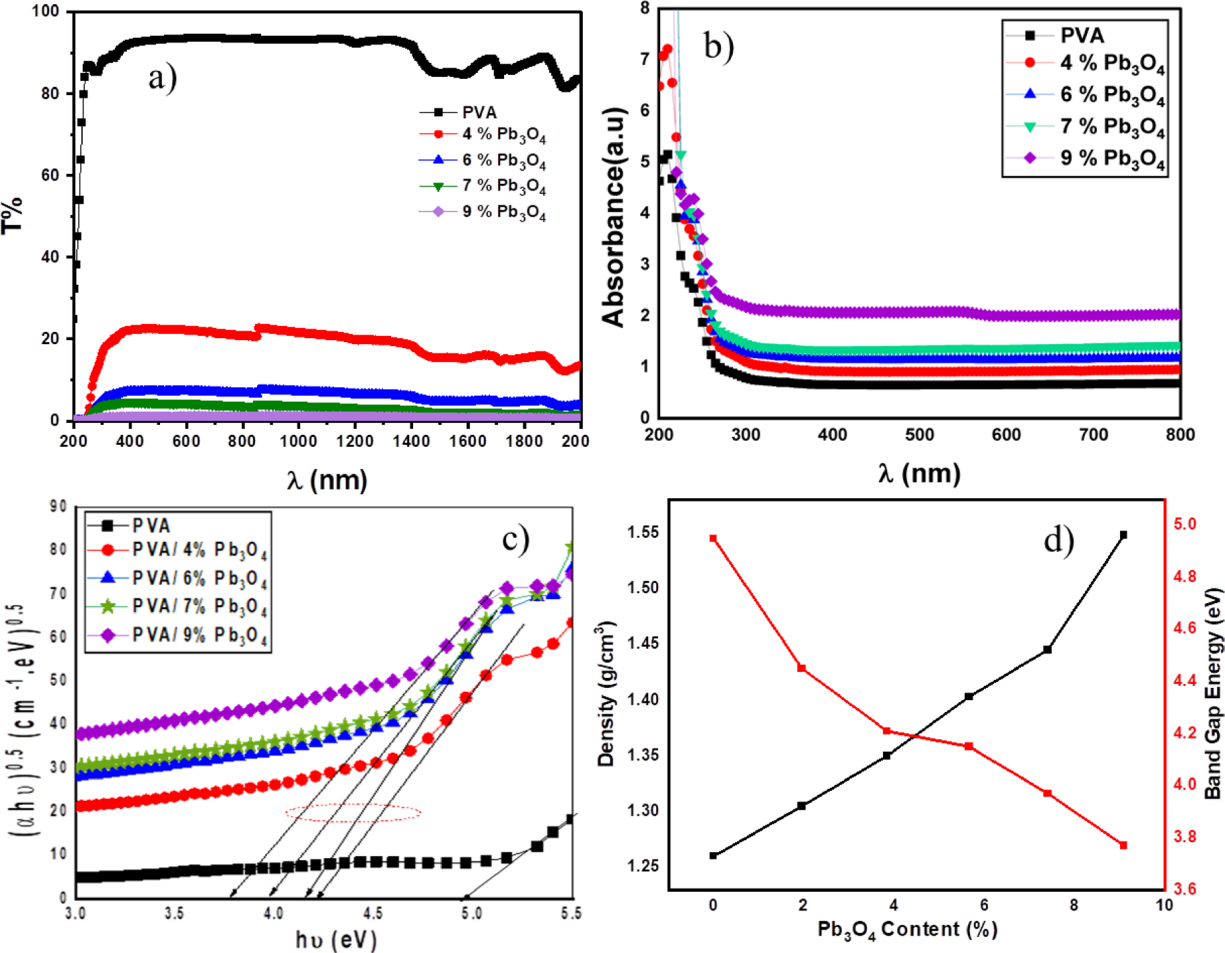
(**a**) The optical transmittance (%), (**b**) The absorbance (a.u) of the prepared samples versus wavelength (nm), (**c**) Tauc-plot analysis for the PVA/Pb₃O₄ films indicating the indirect band-gap transition (s) and (**d**) Variation of the density and Energy band gap with Pb_3_O_4_ content (%).

The optical characteristics of PVA/Pb_3_O_4_ composites demonstrate considerable concentration-dependent variations. Optical transmittance (T %) (see Fig. 4a) across UV–Vis-NIR spectrum (200–2000 nm) significantly diminishes with elevated Pb_3_O_4_loading, declining from approximately 85–90% for pure PVA to below 10% for composites containing 6–9 wt% Pb_3_O_4_, a phenomenon ascribed to the increased density resulting from lead’s high atomic number (Z = 82). In contrast, UV–Vis absorption (200–800 nm) (see Fig. 4b) demonstrates significant UV absorption (λ < 300 nm) with negligible visible-light absorption (λ > 400 nm), maintaining transparency; the incorporation of Pb_3_O_4_ alters absorption intensity without affecting the underlying trend.

Tauc plot analysis^52,53^ (see Fig. 4c) validates indirect allowed transitions, indicating a gradual decrease in optical bandgap (Eg) from 4.95 eV (pure PVA) to 3.77 eV (9 wt% Pb_3_O_4_) as a result of intensified PVA/ Pb_3_O_4_ interaction (see Fig. 4d**)**. This reduction can be attributed to the formation of sub-bandgap states via Pb⁺^2^/Pb⁺^4^ orbitals hybridizing with PVA’s oxygen lone pairs, facilitating charge transfer complexes that lower transition energy barriers. Also, formation of a percolating three-dimensional (3D) networked microstructure^28,54,55^,where Pb_3_O_4_ nanoparticles establish interconnected conductive pathways through electron-hopping mechanisms. The E_g_ concentration inverse proportionality confirms strong PVA/Pb_3_O_4_ electronic coupling, transitioning the composite from insulator to semiconductor while retaining visible transparency, thus enabling dual-functional optoelectronic applications in UV filters and flexible photodetectors.

### Density of PVA/ Pb_3_O_4_ films:

The density of the prepared samples is determined using Archimedes’ principle, with an electric balance employed to measure the weights. The samples are weighed in air (A) and then in toluene (B). The densities of the samples are calculated using Archimedes’ law, expressed in the following Eq. ^56,57^

9 $${\rho }_{S}=\frac{A}{A-B}{\rho }_{T}$$

where, ${\rho }_{S}$ is the experimental density of the sample, ${\rho }_{T}$ is the density of toluene and equal to 0.867g/cm^3^^58^.The densities of samples were found in the range 1.26–1.54 g cm^−3^, as shown in Table S1. Figure 4d shows the density of PVA/ Pb_3_O_4_ nanocomposites film with different Pb_3_O_4_ content. The increased density of the samples is due to the higher atomic number and lead density, which improves the material’s ability to attenuate gamma radiation through interaction mechanisms such as the photoelectric effect and Compton scattering. The inverse correlation indicates that the simultaneous addition of Pb_3_O_4_ increases the radiation shielding efficiency by increasing the density and improves the optical properties of the polymer matrix, as previously reported ^33,59^.

### Shielding parameters

#### Linear attenuation coefficient (LAC)

Table 1 presents the linear attenuation coefficients (LACs) of PVA samples with varying percentages of Pb_3_O_4_, measured at specific energy levels from 0.511 to 1.332 MeV. The LAC values ranged from 0.148 ± 0.007 for sample S1 (0% Pb_3_O_4_) at 1.332 MeV to 0.336 ± 0.018 for sample S6 (9% Pb_3_O_4_) at 0.511 MeV. Sample S1 has the lowest LAC values across all energy levels, indicating that the lack of Pb_3_O_4_ reduces its attenuation capability. As the concentration of Pb_3_O_4_ increases from S1 (0% Pb_3_O_4_) to S6 (9% Pb_3_O_4_), the LAC values rise at each energy level, demonstrating that higher Pb_3_O_4_ content improves the PVA’s ability to attenuate gamma radiation. For example, at 1.332 MeV, the LAC for S1 is 0.148 ± 0.007 cm⁻^1^, while it increases to 0.202 ± 0.010 cm⁻^1^ for S6, reflecting a 36% increase^7^.

**Table 1:** The linear attenuation coefficients (cm^-1^) of the prepared polymeric composites at different gamma energies.

Samples	Linear attenuation coefficient (cm^**-1**^)				
	0.511 MeV	0.662 MeV	1.173 MeV	1.275 MeV	1.332 MeV
PVA	0.235 ± 0.011	0.205 ± 0.010	0.161 ± 0.008	0.155 ± 0.008	0.148 ± 0.007
PVA/2% Pb_3_O_4_	0.253 ± 0.018	0.219 ± 0.016	0.169 ± 0.0112	0.163 ± 0.012	0.157 ± 0.011
PVA/4% Pb_3_O_4_	0.271 ± 0.019	0.237 ± 0.017	0.179 ± 0.013	0.171 ± 0.012	0.168 ± 0.012
PVA/6% Pb_3_O_4_	0.289 ± 0.023	0.254 ± 0.020	0.190 ± 0.015	0.180 ± 0.015	0.176 ± 0.014
PVA/7% Pb_3_O_4_	0.307 ± 0.022	0.266 ± 0.019	0.199 ± 0.014	0.188 ± 0.014	0.187 ± 0.013
PVA/9% Pb_3_O_4_	0.336 ± 0.018	0.294 ± 0.015	0.216 ± 0.011	0.206 ± 0.011	0.202 ± 0.010

This pattern is consistent across all energy levels, indicating a strong correlation between Pb_3_O_4_ concentration and attenuation. The improvement in shielding properties with the addition of lead is attributed to its high density and large atomic number (Z = 82), which increases the interaction of gamma photons with the sample, leading to enhanced attenuation. The primary ways gamma rays interact with matter include the photoelectric effect, Compton scattering, and pair production^36^.

**Fig. 5:**
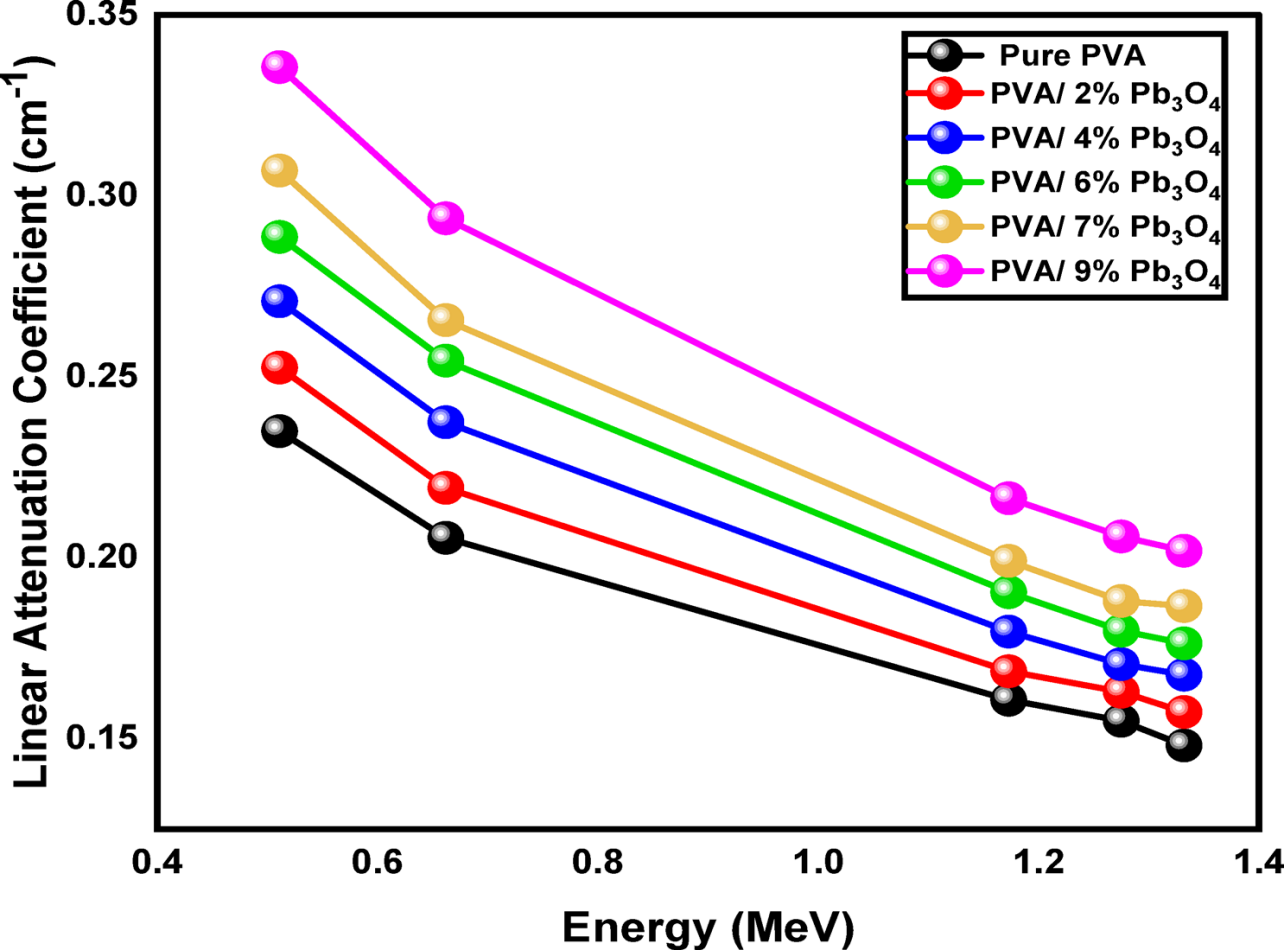
Dependence of linear attenuation coefficient of the samples on gamma ray energy.

Figure 5 illustrates the relationship between linear attenuation coefficients (cm⁻^1^) and gamma-ray energy (MeV). The graph clearly demonstrates an inverse relationship between the linear attenuation coefficients and gamma-ray energy across all measured samples. This observed inverse relationship aligns with established radiation interaction models and can be explained by the connection between photon energy and interaction mechanisms. At lower energies, gamma photons primarily interact with matter through photoelectric absorption and Rayleigh scattering, which are more significant at these energy levels, leading to higher attenuation. As photon energy increases, the main interaction mechanism shifts to Compton scattering, where photons collide with electrons and scatter instead of being absorbed, resulting in reduced energy loss to the material^60^.

#### Mass attenuation coefficient (MAC)

To validate the experimental results, mass attenuation coefficients (MACs) are calculated both experimentally using Eq. 4 and theoretically through the Phy-X software. The chemical compositions of each PVA sample were input into the Phy-X program to obtain the theoretical data^38^. The results from the experimental MACs (using Eq. 4) and those from the Phy-X software are presented in Table 2. The experimental findings showed strong agreement with the calculations from Phy-X, with deviations between their values not exceeding 4%, as in Table 2. This deviation is within the accepted experimental limits and can be attributed to: variations in sample thickness uniformity (± 3 μm) and uncertainty in density measurement (~ 2%) using the Archimedes method; variations in the distribution of microscopic nanoparticles from theoretical uniformity; measurement setup factors, including detector geometry and counting statistics; and limitations of the Phy-X software in considering nanoscale effects in composite materials.

**Table 2:** Experimental and Phy-X of the Mass Attenuation Coefficients (cm^2^/g) of the prepared polymeric composites at different gamma energies.

Energy (MeV) Samples	Mass attenuation coefficient (cm^2^/g)								
	0.511 MeV			0.662 MeV			1.173 MeV		
	Phy-x	Exp	Dev (%)	Phy-x	Exp	Dev (%)	Phy-x	Exp	Dev (%)
PVA	0.188	0.186 ± 0.014	1.006	0.168	0.163 ± 0.012	3.127	0.128	0.128 ± 0.010	0.504
PVA/2% Pb_3_O_4_	0.194	0.194 ± 0.023	0.427	0.173	0.168 ± 0.021	2.660	0.131	0.129 ± 0.016	1.117
PVA/4% Pb_3_O_4_	0.200	0.201 ± 0.026	0.159	0.177	0.176 ± 0.023	0.583	0.133	0.133 ± 0.017	0.092
PVA/6% Pb_3_O_4_	0.206	0.206 ± 0.032	0.294	0.181	0.181 ± 0.028	0.095	0.136	0.136 ± 0.021	0.115
PVA/7% Pb_3_O_4_	0.212	0.212 ± 0.032	0.057	0.185	0.184 ± 0.029	0.879	0.138	0.135 ± 0.021	0.187
PVA/9% Pb_3_O_4_	0.218	0.217 ± 0.025	0.696	0.190	0.190 ± 0.022	0.023	0.141	0.140 ± 0.016	0.490

Energy (MeV) Samples	Mass attenuation coefficient (cm^2^/g)					
	1.275 MeV			1.332 MeV		
	Phy-x	Exp	Dev (%)	Phy-x	Exp	Dev (%)
PVA	0.123	0.123 ± 0.010	0.071	0.120	0.118 ± 0.009	2.106
PVA/2% Pb_3_O_4_	0.125	0.125 ± 0.015	0.234	0.122	0.121 ± 0.015	1.407
PVA/4% Pb_3_O_4_	0.127	0.126 ± 0.017	0.867	0.125	0.124 ± 0.016	0.289
PVA/6% Pb_3_O_4_	0.130	0.128 ± 0.020	1.242	0.127	0.126 ± 0.018	0.868
PVA/7% Pb_3_O_4_	0.132	0.130 ± 0.020	1.503	0.129	0.129 ± 0.019	0.080
PVA/9% Pb_3_O_4_	0.134	0.133 ± 0.016	1.047	0.131	0.130 ± 0.015	0.656

From Table 2, it can be seen that PVA samples (S1-S6) exhibit their highest attenuation capability at 0.511 MeV, which corresponds to the higher MACs at this energy. The MAC values range from 0.186 ± 0.014 to 0.217 ± 0.025 cm^2^/g at 0.511 MeV. However, the attenuation capability of the PVA samples diminishes at higher energies, becoming less effective at 1.332 MeV, where the minimum MAC is recorded. The data in Table 2 also indicates a positive correlation between the addition of Pb_3_O_4_ and the attenuation capability of PVA, with S6 showing a higher MAC than samples S1-S5 at the examined energy levels.

**Fig. 6:**
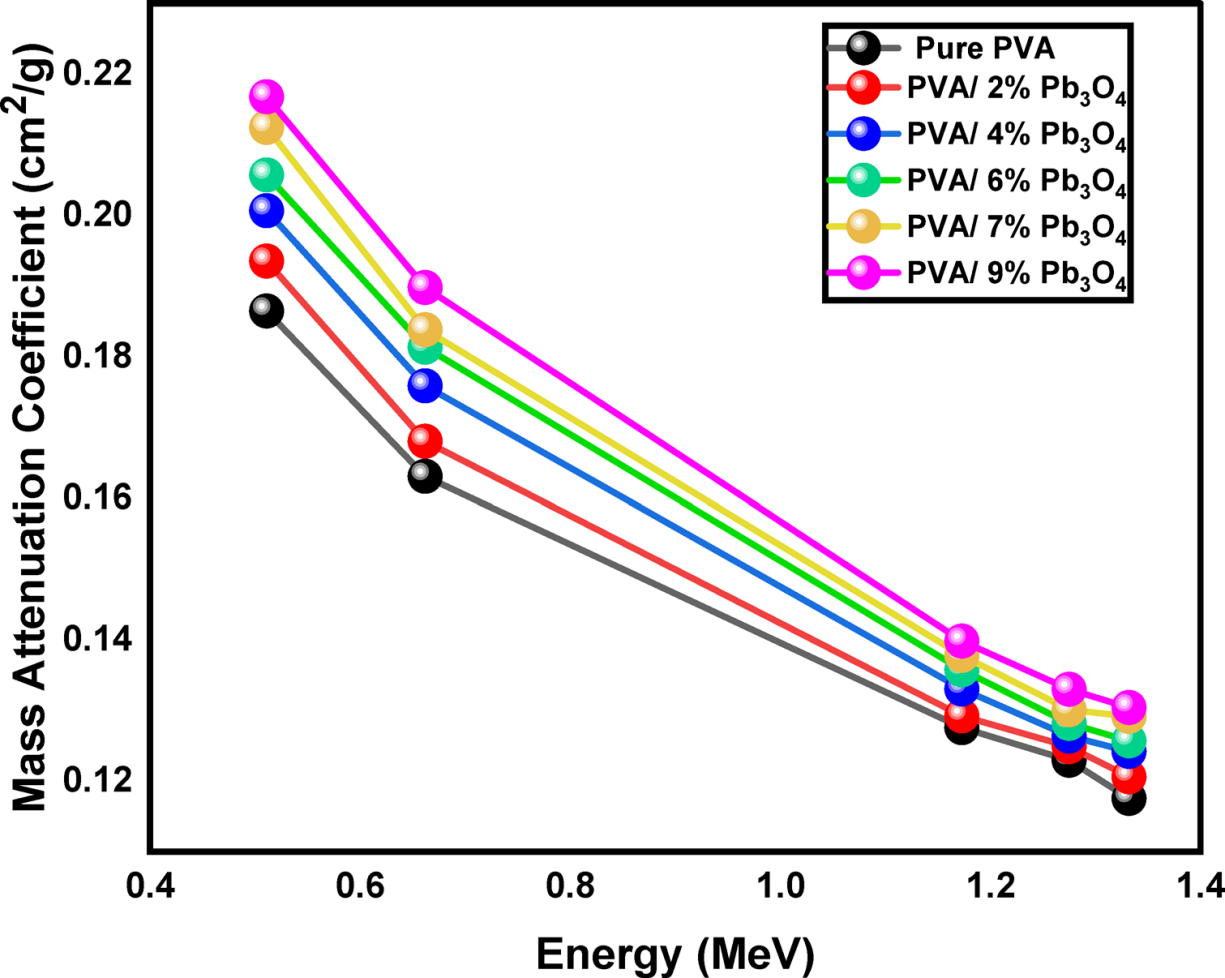
Variation of the mass attenuation coefficient of the samples with gamma ray energy (MeV).

Figure 6 illustrates that the mass attenuation coefficients decline as photon energy increases, reflecting a reduced probability of photon interactions at higher energies. Consequently, no significant differences in mass attenuation coefficients are observed at elevated photon energies. Conversely, at lower energies, the mass attenuation coefficients increase with higher concentrations of Pb_3_O_4_^61^. The findings indicate that all interaction types decrease with rising photon energy, which corresponds to the observed decline in mass attenuation coefficients for the PVA samples. Additionally, they demonstrate an increased probability of interactions with higher Pb_3_O_4_ concentrations across all interaction types^34,62^.

Table 3 presents the comparison of the energy gap (E_g_) and MAC values of other polymer composite systems^63–70^ and the present study’s values. This comparison demonstrates the better excellence of the synthesized (PVA/Pb_3_O_4_) composite. It provided the maximum value of the mass attenuation coefficient (0.190 cm^2^/g), indicating its high radiation shielding property. Most notably, this high performance was achieved at a very low concentration of filler of just 9%, compared to the other materials which required concentrations as high as 50% in order to achieve less effective effects. Also, its relatively low energy gap of 3.77 eV suggests excellent optical and electronic properties, demonstrating it to be an extremely efficient and useful material.

**Table 3:** Energy gap and mass attenuation coefficient values for different polymer composite systems.

Composites	Filler (wt %)	Eg (eV)	MAC at 0.662 MeV (cm^2^/g)		References
PVA/NaSe	18.5	4.685	–	^63^	
PVA/ BaTiO3	3.57	5.6	–	^43^	
PVA/ Al2O3	1–3	4.89	–	^64^	
PVA-PVP/ MgO	6.25	4.02	–	^65^	
PVC/ BiVO4	6	4.06	0.085	^66^	
PVA/ Er + 3 and Dy + 3	30	5.16	–	^24^	
PVA/ VCl3	18.25	3.03	–	^41^	
PVA/ CuO	12	3.18	–	^53^	
PVA:PbO2	0.2	4.33	–	^44^	
PVA/ WO3	50	–	0.059	^67^	
PVA/ Bi2O3and Na2O4W	50	–	0.097	^68^	
PVA / Bi₂O₃	12%	3.55	–	^69^	
PVA / PbO	1.5 g PbO	–	0.169	^34^	
PVA / Bi₂O₃	50%	–	0.096	^70^	
PVA / Pb3O4	9%	3.77	0.190	Current study	

#### Half value layer (HVL) and mean free path (MFP)

**Fig. 7:**
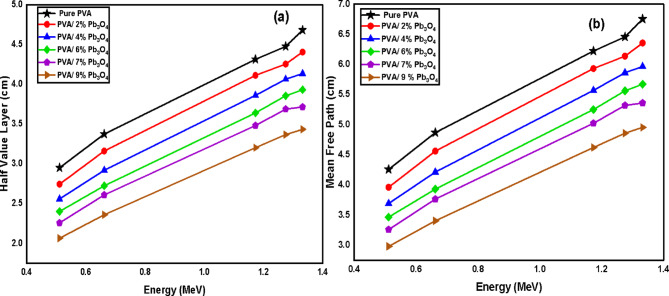
Variation of (**a**) the half value layer (cm) and (**b**) mean free path of the polymeric samples with the energy of gamma ray photons.

Other shielding parameters, namely HVL and MFP, are determined using Eqs. (6, 7). The resulting data presented in Table S2 and Fig. 7. The findings indicate a significant impact of adding Pb_3_O_4_ nanocomposite on both HVL and MFP. Sample S1 (0 wt % Pb_3_O_4_), exhibits the highest HVL and MFP values at all energies, ranging from 2.950 to 4.256 cm at 0.511 MeV to 4.680 and 6.752 cm at 1.332 MeV, respectively. As the concentration of Pb_3_O_4_ increases in samples S2 through S6, both HVL and MFP generally decrease, indicating improved attenuation capabilities due to the presence of Pb_3_O_4_ particles.

From the HVL data, it is clear that the thickness required for pure PVA composites is significantly greater than that for PVA composites with added Pb_3_O_4_ to achieve 50% gamma ray attenuation for energies below 1.332 MeV. For instance, at 0.511 MeV, the pure PVA composite needs approximately 3 cm of thickness to halve the gamma ray intensity, while the composite with 9 wt% Pb_3_O_4_ requires only 2 cm. This suggests that PVA composites containing Pb_3_O_4_ demonstrate superior gamma shielding capabilities compared to pure PVA in the lower energy range^24^.

Regarding the MFP data, the lowest MFP is observed at the lowest energy measured (0.511 MeV), while the highest MFP occurs at 1.332 MeV. This trend indicates that higher energy radiation can penetrate the material more easily. A denser material results in more interactions between photons and atoms, leading to greater attenuation. Thus, the material’s density affects the likelihood of radiation interacting with it. Throughout all studied energies, PVA composites with Pb_3_O_4_ show the lowest MFP values, reflecting an increase in photon interactions. For example, the composite with 9 wt % Pb_3_O_4_ has MFP values of 2.980 and 4.953 cm at 0.511 and 1.332 MeV, respectively, compared to the pure PVA values of 4.256 and 6.752 cm at the same energies. This highlights the importance of atomic distribution in influencing the number of photon interactions^36^.

## Scalability and environmental safety considerations

Significant health and environmental concerns associated with lead-containing materials are mitigated by encapsulating Pb₃O₄ nanoparticles within a PVA matrix. To improve quality, the casting method is advantageous for the following reasons: (a) aqueous processing eliminates the risk of organic solvents, (b) room-temperature processing reduces energy consumption, and (c) the method is compatible with roll-to-roll processing technologies. From an environmental perspective, SEM/EDX also confirmed that the complete encapsulation of Pb₃O₄ within the polymer matrix prevents the emission of nanoparticles under normal operating conditions. There is also a reduced potential for occupational exposure during manufacturing. Composite films can be safely destroyed thanks to certified hazardous waste disposal processes. Furthermore, these composite films reduce the need for replacement and maintenance costs due to their long service life. Continuous monitoring of film thickness and chemical composition during manufacturing ensures consistent performance characteristics with minimal material loss and controlled manufacturing quality.

## Conclusions

This current study evaluated the effect of incorporating Pb₃O₄ NPs at concentrations ranging from 0 to 9 wt% into PVA films, fabricated via the solution casting method, on their structural, optical, and gamma-ray shielding properties. XRD analysis proved enhanced crystallinity with increasing Pb₃O₄ content. At the same time, optical characterization showed a significant reduction in transmittance (from 85 to 90% for pure PVA to < 10% for 9 wt% Pb₃O₄) and a decreased bandgap energy from 4.95 to 3.77 eV, indicating improved optoelectronic potential. Gamma ray shielding performance, examined at energies of 0.511–1.332 MeV, showed superior attenuation with higher Pb₃O₄ concentrations, with the PVA/9% Pb₃O₄ film achieving the highest linear attenuation coefficient (0.336 ± 0.018 cm⁻^1^ at 0.511 MeV) and mass attenuation coefficient (0.190 cm^2^/g at 0.662 MeV), performed better than other polymer composites with lower filler content. The half-value layer (HVL) and mean free path (MFP) decreased significantly, with the 9 wt% Pb₃O₄ composite requiring only ~ 2 cm thickness to halve gamma ray intensity at 0.511 MeV compared to ~ 3 cm for pure PVA. Encapsulation of Pb₃O₄ within the PVA matrix effectively mitigated its toxicity, enhancing biosafety for medical and industrial applications. Experimental results aligned closely with theoretical predictions from Phy-X/PSD software, with deviations < 4%. These findings proved PVA/Pb₃O₄ nanocomposite films as a lightweight, flexible, and highly effective alternative to traditional heavy shielding materials for gamma ray shielding in medical, industrial, and nuclear research applications.

## Supplementary Information

Below is the link to the electronic supplementary material.

Supplementary Material 1

## Data Availability

This manuscript has data associated with it in a data repository. [Authors’ comment: All data included in this manuscript are available upon request by contacting the corresponding author].
