# Supplementary material for: Effect of Pb3O4 nanocomposite on the structural, optical, and radiation shielding properties of PVA Films
Source: Sci Rep. 2025 Oct 31;15:38104. doi: 10.1038/s41598-025-22740-6 (PMC12578903; doi:10.1038/s41598-025-22740-6)
Supplement: Supplementary file 1 — Supplementary Material 1 [file 41598_2025_22740_MOESM1_ESM.pdf]

Supplementary material

Effect of Pb_3O_4 nanocomposite on the structural, optical, and radiation shielding properties of PVA Films

Yasmin Hamed [a*], Khaled Salahel Din [a], S. Harb[a], Sahar Elnobi [a*]

[a] Physics Department, Faculty of Science, South Valley University, Qena 83523, Egypt

*Corresponding author,

Yassmein.Mohamed@sci.svu.edu.eg **(Yasmin Hamed)**

Sahar.elnobi@sci.svu.edu.eg **(Sahar Elnobi)**

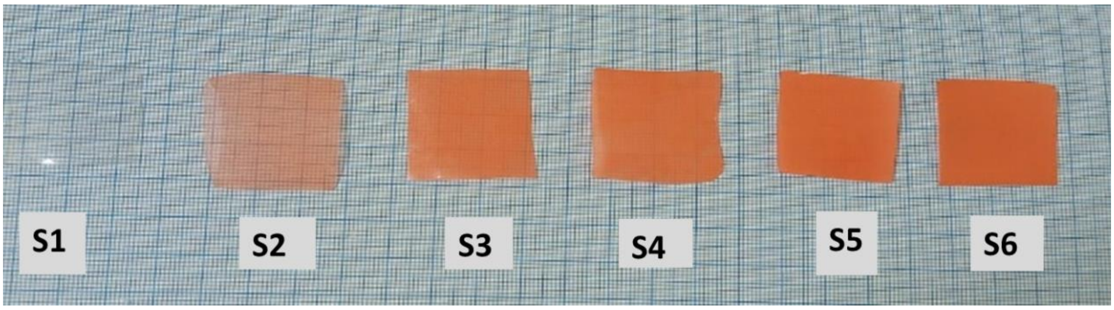

Schematic S1. The PVA/Pb_3O_4 samples.

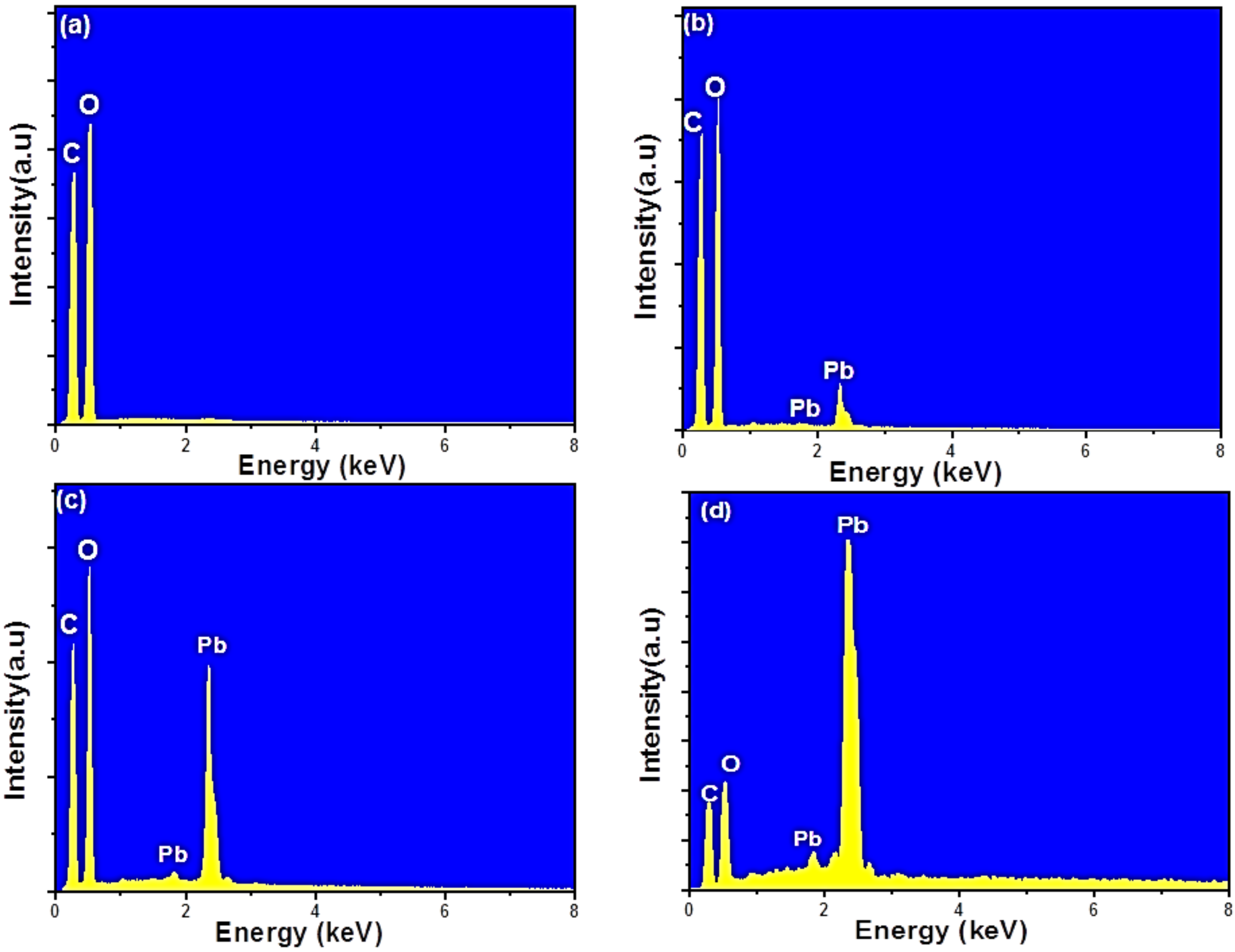

Fig. S1: EDX spectra (a) pure PVA, (b) PVA/2%Pb_3O_4, (c) PVA/6%Pb_3O_4, and PVA/9%Pb_3O_4.

Table S1: Concentration of Pb_3O_4 and density (g/cm^3) of PVA/Pb_3O_4 samples

Samples	Pb_3O_4 (wt. %)	Density (g/cm^3)
PVA	0	1.26±0.010
PVA/2% Pb_3O_4	2	1.31±0.002
PVA/4% Pb_3O_4	4	1.35±0.010
PVA/6% Pb_3O_4	6	1.40±0.002
PVA/7% Pb_3O_4	7	1.45±0.003
PVA/9% Pb_3O_4	9	1.55±0.002

Table S2: Half value layer and mean free path of the prepared polymeric composites at different gamma ray energies

Samples	0.511 MeV		0.662 MeV		1.173 MeV		1.275 MeV		1.332 MeV	
	HVL (cm)	MFP (cm)	HVL (cm)	MFP (cm)	HVL (cm)	MFP (cm)	HVL (cm)	MFP (cm)	HVL (cm)	MFP (cm)
PVA	2.950	4.256	3.374	4.867	4.312	6.221	4.475	6.456	4.680	6.752
PVA/2% Pb_3O_4	2.745	3.960	3.161	4.561	4.111	5.930	4.254	6.137	4.405	6.355
PVA/4% Pb_3O_4	2.559	3.692	2.920	4.212	3.860	5.569	4.063	5.862	4.134	5.965
PVA/6% Pb_3O_4	2.402	3.465	2.724	3.930	3.639	5.251	3.855	5.561	3.931	5.671
PVA/7% Pb_3O_4	2.258	3.258	2.609	3.765	3.481	5.022	3.687	5.319	3.715	5.359
PVA/9% Pb_3O_4	2.066	2.980	2.359	3.404	3.202	4.620	3.367	4.857	3.433	4.953
